# A description of the development and initial evaluation of a patient navigator delivered patient portal enrollment program

**DOI:** 10.1016/j.pec.2025.109343

**Published:** 2025-09-08

**Authors:** Alicia K. Matthews, Caleb Gumbs, Larisa Burke, Brittany Harris Vilona, Alejandra Rodriguez, Hope Opuada, Kristina Rowden, Ezra Root, Rachel Caskey

**Affiliations:** a School of Nursing, Columbia University, New York, USA; b Department of Medicine, University of Illinois Chicago, Chicago, USA; c College of Nursing, University of Illinois Chicago, Chicago, USA

**Keywords:** Patient Portal Enrollment, Patient Navigators, Federally Qualified Health Centers, Digital Health Disparities

## Abstract

**Objectives:**

Uptake of electronic patient portals is low in underserved populations. Federally qualified health centers (FQHCs) face unique barriers, including limited digital literacy, language differences, and low trust in technology. This paper describes the development and initial evaluation of a patient navigator program aimed at increasing portal enrollment in three FQHC locations.

**Methods:**

Development and implementation followed a seven-step process guided by the Knowledge to Action Framework: (1) stakeholder engagement to identify barriers, (2) literature review to contextualize disparities, (3) resource and workflow assessment, (4) stakeholder feedback to refine the navigator approach, (5) a monitoring system to track enrollment and navigator activities, (6) identification of structural barriers and sustainability strategies, and (7) project evaluation. Navigators were embedded in clinic workflows to assist with MyChart enrollment, provide technical help, and address concerns. Activation rates were assessed with pre-post comparisons and navigator tracking data.

**Results:**

Tailored materials, including a MyChart handout and a smaller flyer for login details, were created. Over 12 months, of 1658 eligible patients, 1062 (64 %) accepted navigator assistance; 790 (74 %) successfully activated MyChart. African American (44 % to 49 %) and Latinx (52 % to 60 %) patients showed notable gains, and nearly all (99 %) newly enrolled users accessed MyChart at least once post-activation.

**Conclusions:**

Embedding navigators proved feasible for increasing MyChart enrollment in FQHC settings. The structured process highlighted essential considerations for scalability. Future work should examine long-term sustainability and how patient navigation affects broader engagement and health outcomes.

**Practice implications:**

By offering real-time, culturally attuned support, navigators can help patients overcome digital literacy barriers, thereby improving enrollment in patient portals. Health systems seeking to reduce disparities may benefit from adopting navigator-led strategies as part of routine care.

## 1. Introduction

Telehealth and electronic health records (EHRs) have grown exponentially in recent years, especially following the onset of the COVID-19 pandemic [1,2]. The rapid expansion of telehealth and other electronically mediated health delivery systems has transformed healthcare delivery by reducing the need for in-person visits and increasing access to medical information and services [1,2]. Despite these technological advancements, disparities in digital health engagement persist, particularly among older individuals, those with lower digital literacy, and people living in lower socioeconomic contexts [3–5]. Patient portals address these disparities by allowing patients to access their electronic health records, schedule appointments, view lab results, and communicate directly with providers through secure messaging. However, enrollment in and usage of portals has been limited.

Many healthcare organizations have sought targeted strategies to increase portal uptake in response to the ongoing public health concern about portal underutilization among underserved populations. These strategies have included simplifying the enrollment processes and procedures, emphasizing the potential benefits of portal use, and providing additional support for patients when needed [6,7]. Even with these measures in place at many centers, patient enrollment rates have been found to remain suboptimal. This notable disparity suggests that only offering portal access is insufficient when working toward equitable digital access. Researchers have found that socioeconomic and linguistic barriers and a lack of trust in technology further complicate portal engagement among populations that could benefit significantly from immediate access to health information [6,8]. Identifying opportunities and strategies for evidence-based interventions that can address these challenges in Federally Qualified Health Center (FQHC) contexts is critically important as barriers are often ever-present for patients of these centers.

Patient navigation is an approach that has proven effective in enhancing healthcare access, adherence to screening recommendations, and linkage to care for diverse patient populations [9,10]. Patient navigators may play a crucial role in the context of the patient portal program. They guide and support patients through the healthcare system to facilitate access to care, improve health outcomes, and enhance patient experience. This includes assisting with scheduling appointments, understanding medical information, overcoming barriers to care, and coordinating services among healthcare providers [11]. Patient navigators offer in-person, hands-on support, which is more effective than passive interventions like pamphlets or online tutorials [12]. Many underserved patients distrust digital health tools due to historical healthcare discrimination and low technological confidence [7]. Navigators, often sharing similar backgrounds, help bridge trust gaps by providing culturally tailored explanations [13]. In many settings, navigators have guided patients through complex healthcare systems, provided culturally relevant health education, and reduced structural barriers that hinder the use of preventive services [9,10,12,13]. Integrating patient navigators to facilitate patient portal enrollment and utilization can help reduce digital and health literacy gaps by offering hands-on, personalized support. This approach may be particularly relevant to FQHCs, which serve communities with multiple comorbidities and limited resources.

To address these disparities, we collaborated with Mile Square Health Center (MSHC) to implement a patient navigation strategy to enhance patient portal engagement. Navigators were embedded in clinic workflows to assist patients without an active portal account, helping them register, learn portal functionality, and troubleshoot technical issues. The goal of this initiative was to develop a replicable strategy for increasing portal enrollment in community settings with known digital and health literacy barriers. This paper describes the methods used to implement the program and presents initial evaluation results illustrating changes in portal enrollment and usage following its introduction.

## 2. Methods

### 2.1. Study design and setting

This study was part of the NIH-funded MiQuit Care initiative, which focused on improving access to digital health tools among underserved populations. It was conducted at MSHC, an FQHC network affiliated with an academic medical center in Chicago that primarily serves low-income patients, including many racial and ethnic minority groups with public insurance. Patient navigators were bachelor’s degree–prepared, with academic backgrounds in public health or related fields, and received project-specific training in patient portal enrollment and digital health literacy support. They assisted patients with MyChart, the patient portal linked to Epic, one of the most widely used electronic health record (EHR) systems in the United States. The inclusion criteria for program participation were developed and reviewed by the MiQuit Care study team, including clinical leadership and health services researchers, and approved by the institutional review board. Demographic information and MyChart activation trends were tracked before and after the introduction of navigators. All study activities were approved by the University of Illinois Chicago Institutional Review Board (IRB #2020–0532 & 2021–0578).

The Knowledge-to-Action (KTA) Framework, developed by Graham et al. [14], was a foundational guide for developing and implementing the patient portal enrollment approach. This framework bridges research evidence and practical application in real-world settings. It consists of two key cycles: 1) Knowledge creation, which involves gathering and adapting evidence to fit the local context, and 2) Action cycle, which focuses on implementing, monitoring, and sustaining the intervention. Central to the KTA Framework is stakeholder engagement, which ensures that the approach remains relevant and responsive to the identified challenges throughout the process. We followed the following seven steps to guide the development and evaluation of our patient portal navigation program.

### 2.2. Action step 1: Initial stakeholder engagement

Data was collected during this study as part of a larger project to develop a patient portal–delivered intervention for low-income populations. Patient interviews and data collection took place between August and October 2021. The research used a qualitative study design that evaluated the implementation of a patient portal–focused intervention for low-income patients at MSHC. The patient navigators at Mile Square recruited a volunteer sample of patients through various outreach methods, such as doctor referrals and face-to-face approach techniques, and posted flyers in the clinic. Every patient had to fulfill a set list of eligibility requirements, including being 21 years or older, being a patient at Mile Square, being English-speaking, and being able to provide informed consent [8]. Once the patients were eligible and approved to complete an interview, the participants were asked to complete a less than 10-minute self-administered survey documenting their demographics and attitudes toward patient portal use. Before each interview began, whether conducted over the phone, via Zoom, or in person, the questionnaire data responses were entered into REDCap, an electronic research database.

After data collection, participants completed structured qualitative interviews to explore their experiences with patient portals. The process included rapport-building, guided discussions, audio recordings, and post-session debriefing to highlight key findings [8]. The Practical, Robust Implementation and Sustainability Model (PRISM) helped frame the study, focusing on how interventions are adopted and sustained in healthcare settings [15]. Interviewers used PRISM to better understand patient perspectives, identifying barriers and facilitators to portal use. This approach ensures that digital health strategies are practical, equitable, and responsive to patient needs.

In the interviews, the participants discussed their understanding of patient portals, barriers impeding enrollment, perceived benefits, and recommended strategies for increasing adoption [8]. Overall, patients and providers exhibited gaps in knowledge regarding the portal’s core features, namely, secure messaging with providers, viewing lab results, and scheduling appointments. Providers also noted that limited clinic time often hindered efforts to familiarize patients with portal functionalities. Despite these challenges, the participants acknowledged multiple advantages, including enhanced patient-provider communication and more convenient access to health information. Some commonly reported barriers included low digital literacy, privacy concerns, and a preference for in-person interactions. Many responses emphasized hands-on enrollment support, user-friendly educational materials tailored to low-literacy audiences, and consistent staff training as promising ways to address knowledge gaps and expand patient portal usage [8].

### 2.3. Action step 2: Review the literature to identify medically underserved patient populations and compare results to the local context

The literature on patient characteristics associated with enrollment in patient portals highlights several key factors influencing portal usage. Age, gender, race, and socioeconomic status are significant predictors of portal use, with younger patients, females, and individuals with higher income and education levels being more likely to enroll. Older adults, racial/ethnic minorities, and individuals with lower socioeconomic status tend to exhibit lower enrollment and usage rates [3–5]. These differences often stem from disparities in digital literacy, technology access, and trust levels in healthcare systems [6,7]. To understand how these broader trends appeared in our local setting, we analyzed 40,852 eligible patients within the MSHC network, finding that approximately 34 % had activated their MyChart accounts. Activation rates were higher among younger adults, females, and those with private insurance, while older adults, racial/ethnic minorities, and those with public insurance had lower rates. These findings emphasized the need for an intervention like patient navigation to address these barriers.

### 2.4. Action step 3: Identify existing physical resources (office space, staff advocates, EHR systems) to create clinic workflow

Workflow refers to the procedural aspect of a working system and focuses on how work activities unfold over time [16]. These properties are crucial because they enable users to utilize tools and access information during critical activities. In Mile Square, where the navigation study was conducted, several aspects of the clinic were vital in ensuring day-to-day activities ran smoothly and efficiently. Mile Square features four nursing stations that can accommodate about forty nurses, three provider stations that can host approximately twenty providers, thirty-three patient consulting rooms, four labs, and four washrooms, with specific provisions for nursing mothers. This setup facilitated seamless interactions and ensured that all necessary administrative and clinical tasks had the physical infrastructure required for efficient operations. Additionally, MyChart is integrated into the Mile Square Health System’s electronic health record, providing a convenient and secure way for patients to manage their healthcare needs, access test results, schedule appointments, and communicate with providers.

**Fig. 1.**
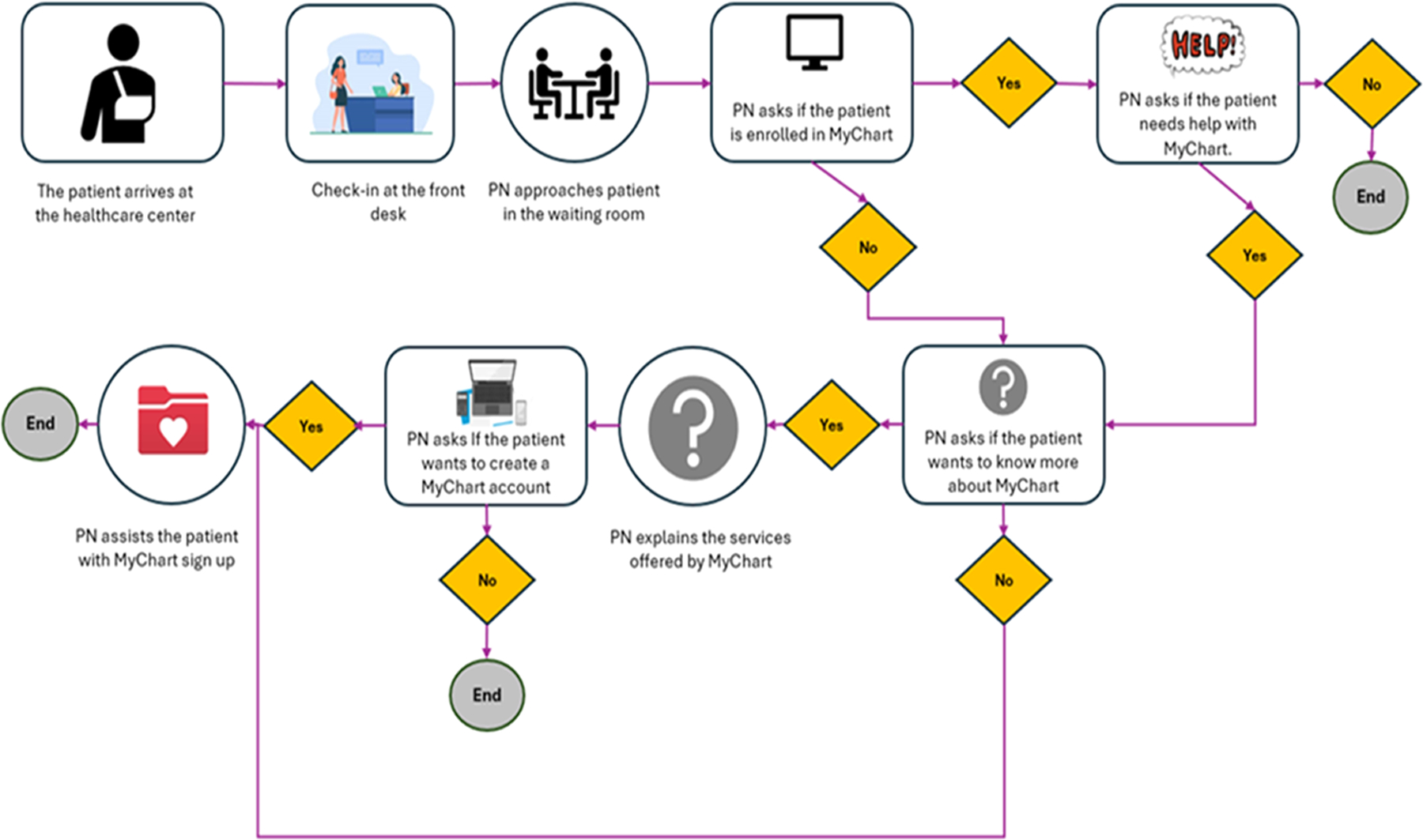
Patient Navigator Clinic Workflow for Patient Portal Enrollment. Abbreviations: PN – Patient Navigator; MyChart – Epic’s patient portal platform.

Fig. 1 displays the workflow for our patient portal enrollment approach. The patient portal enrollment program, led by patient navigators (PNs), streamlines MyChart activation and support within clinic workflows. The process starts when a patient arrives at the healthcare center and checks in at the front desk. A PN then approaches the patient in the waiting room to ask if they are already enrolled in MyChart. If they are enrolled, the PN checks whether they need assistance with the usage of MyChart. If help is needed, the PN provides support (e.g., resetting a password); otherwise, the process ends. If the patient is not enrolled, the PN asks if they want to create a MyChart account. If they agree, the PN assists with the sign-up process. If the patient declines, the process concludes. For patients unfamiliar with MyChart, the PN explains its services and benefits before asking again if they would like to enroll. The PN facilitates account creation if they sign up after the explanation. If they are not interested, the process ends. This structured approach ensures that patients receive tailored support while integrating MyChart enrollment into routine clinic operations.

### 2.5. Action step 4: Further stakeholder engagement to refine processes and materials

The additional engagement was carried out with the patients and healthcare personnel identified in Action Step 1 to tailor the navigation approach to patients with low health literacy and limited digital literacy. Understanding that about 50 % of the U.S. population is affected by low health literacy, the team recognized the need to simplify explanations and ensure patient-friendly language in all communications [12]. Patient navigators were trained to provide patients with information about the patient portal and to provide one-on-one help, which included demonstrating step-by-step logins, clarifying portal functionalities, and addressing remaining questions or confusion. Healthcare personnel feedback also contributed to refining these materials, ensuring that navigators had consistent messaging and a well-defined protocol. For example, the navigators used resources tailored to Mile Square patients’ reading and education levels. These included a MyChart handout, which provides a simple overview of the portal’s services, and a smaller flyer that offers space for patients to record notes about MyChart or their appointments, including login information. Both resources underwent review by multiple providers, staff, and patients to ensure clarity and ease of use.

### 2.6. Action step 5: Identify mechanisms to monitor the patient navigation program implementation and areas needing additional modification

Patient navigators created reports in Epic to identify patients with scheduled appointments at relevant Mile Square clinics. It included their MyChart activation status, clinic location, appointment date/time, and appointment type. Patients without active MyChart accounts were targeted for enrollment. Each scheduled appointment was tracked by the navigator, who classified it as (1) no-show, (2) ineligible (e.g., needed interpreter), (3) declined enrollment, (4) successfully assisted but not activated, (5) successfully activated, (6) assisted but already enrolled, or (7) missed. Monthly summaries of this tracking data allowed the team to assess how many patients were offered assistance and how many activated MyChart. This process also highlighted trends, such as frequent declines or missed opportunities, prompting adjustments in navigator assignments or clinic workflow.

### 2.7. Action step 6: Identify structural barriers to program sustainability and build partnerships

The patient navigation study demonstrated improvements in same-day MyChart activation rates at clinics receiving navigation support [12]. The MSHC leadership pursued ways to sustain and scale the navigation program to build on these improvements. Until Q1 2024, the Mi Quit Care NIH grant was the principal funding source for the navigators. However, the University of Illinois Chicago established a “technology hub” with portal navigation as part of more significant digital initiatives. The Digital Health Hub trains staff to offer personalized support, assisting with issues such as password resets and providing guided instruction on scheduling appointments using MyChart. Mi Quit team members provided training materials and lessons learned regarding best practices to this technology team, anticipating that they would take over enrollment activities. Both teams continued to meet to ensure that the clinics captured all eligible patients, increasing the likelihood that patients left the clinic confident in managing their health from home.

### 2.8. Action step 7: Evaluation of enrollment outcomes

**Fig. 2.**
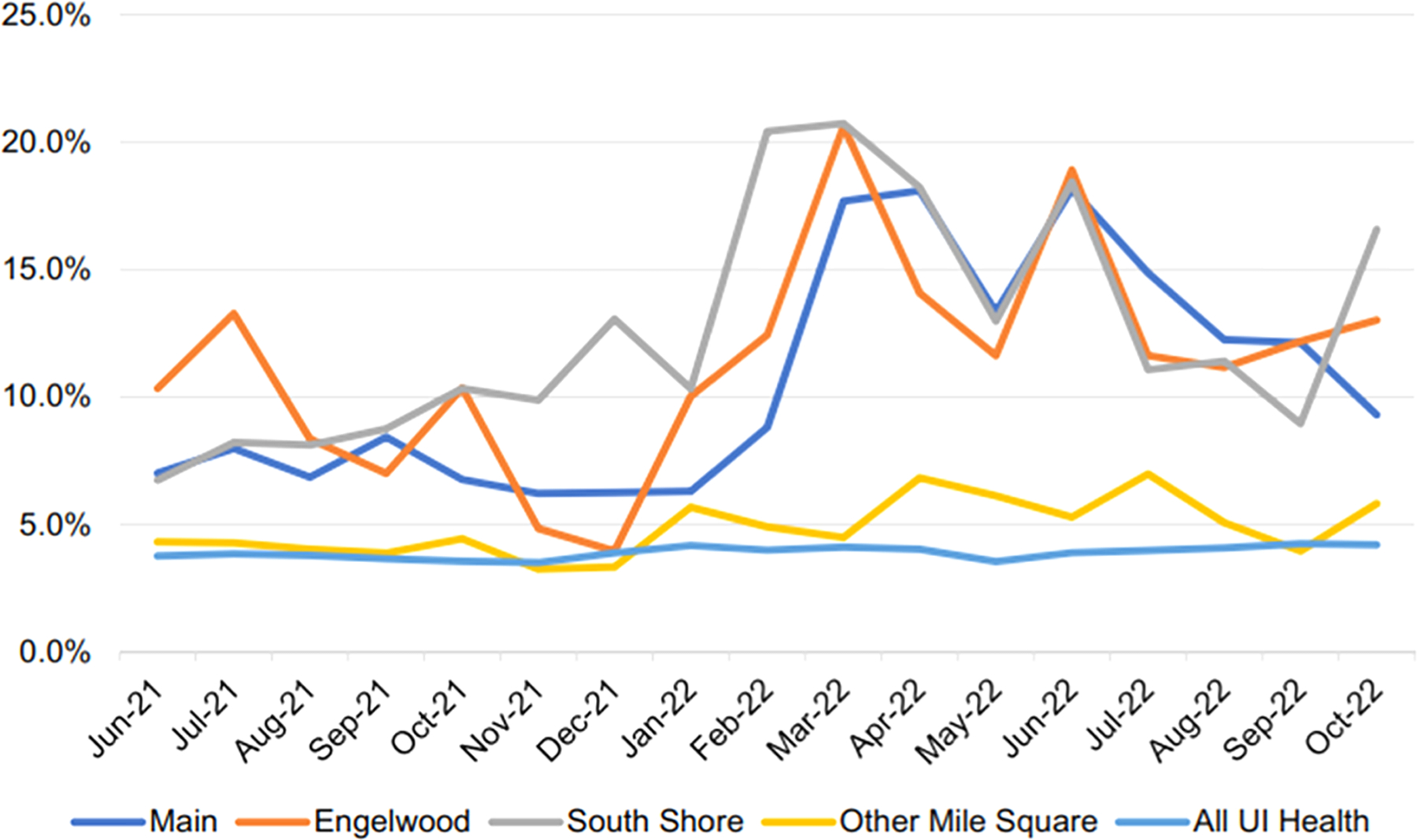
Rates of MyChart Activation for nonactive patients on the same day as the visit [12].

Of 1658 patients eligible for MyChart enrollment across three MSHC clinics (Englewood, South Shore, Main/Urgent Care), 1062 (64 %) accepted navigator support during the study period from June 2021 to October 2022 [12] (Fig. 2). Common reasons for declining included low comfort with technology, lack of internet access, preference for in-person or phone interactions, and not feeling well on the appointment day. Of those who received assistance, 790 (74 %) had successfully activated their MyChart account by the end of their clinic visit. The navigators did not meet with 345 patients (17 %) primarily due to clinic flow concerns, e.g., a provider was with the patient (n = 78, 23 %), the navigator was with another patient (n = 77, 22 %), the patient was scheduled for vaccination only (n = 60, 17 %), the navigator was unavailable (i.e., in a staff meeting) (n = 54, 16 %), the location of the patient was incorrect (n = 48, 14 %), or other reasons such as patient refusal or language barriers (n = 28, 8 %). Navigators also assisted 146 additional patients who were already enrolled in MyChart but experienced barriers in portal use, such as forgotten usernames or passwords.

The success of the patient navigator program is evident in the comparison of MyChart enrollment before and after its introduction. During the pre-navigation phase, 70 % of patients at the three participating clinics were enrolled in MyChart, which rose to 79 % in the post-navigation phase. The proportion of patients who enrolled in their clinic visits increased significantly from 8 % to 14 % in navigator-assisted clinics, compared to a smaller increase from 4 % to 5 % in Mile Square clinics without a navigation program. These results demonstrate the potential of the patient navigator program to increase MyChart enrollment significantly.

Demographic data indicated that the patient population served by these three clinics was predominantly African American (76 %) and female (70 %), with a median age of 42. Medicare or Medicaid covered about 69 % of these patients, reflecting the socioeconomic profile commonly seen in FQHCs. During the navigation period, MyChart activation increased from 44 % to 51 % among this population. There were notable gains among African American patients (44–49 %) and Latinx patients (52–60 %), suggesting that the navigation intervention reached groups typically underrepresented in patient portal use. Nearly all patients who activated MyChart for the first time (99 %) accessed it at least once following account creation.

### 2.9. Discussion and conclusion

Increasingly, healthcare delivery in the United States is linked to technology-based approaches [1,2,17,18]. As such, digital health disparities, particularly among low-income and racial/ethnic minority populations, represent a significant yet modifiable barrier to care. For example, patient portals can potentially improve healthcare access and coordination but are underutilized by underserved communities [3,6]. Studies suggest that provider encouragement and automated reminders can increase portal use [6], but these methods require patients to take independent action. By contrast, patient navigation actively assists patients in real-time, addressing barriers immediately [9,10]. This study evaluated the impact of a patient navigator–delivered intervention on MyChart enrollment at an FQHC network. Community engagement approaches were used to understand patients’ and providers’ knowledge, attitudes, and beliefs about using the patient portal [8,12]. Further, stakeholder input was instrumental in developing patient education materials and establishing the clinic workflow. Evaluation results suggest that patient navigators significantly increased MyChart activation rates among underserved patients [12]. Enrollment rates improved from 70 % to 79 %, with navigator-assisted clinics showing a larger increase (8–14 %) than non-intervention clinics (4–5 %). African American and Latinx patients had notable gains in enrollment (44–49 % and 52–60 %, respectively) [12]. Nearly 99 % of newly activated users accessed MyChart at least once post-enrollment, suggesting that navigators helped patients overcome initial adoption barriers. Previous research on cancer screening navigation programs has shown similar effectiveness in improving patient engagement with healthcare services [10]. These findings underscore the potential of patient navigation in addressing digital health disparities and promoting patient engagement, particularly among underserved populations.

Despite the program’s success, several challenges were identified. The first set of challenges relates to patient barriers. Patients expressed varying levels of interest in and trust in patient portals. Some patients described concerns about the security of their personal health information as a reason for not enrolling in the patient portal. Additionally, some patients preferred in-person or phone-based healthcare interactions, consistent with prior findings [19]. Although navigators assisted with sign-ups, broader socioeconomic factors may still impact ongoing portal usage [3]. For example, many patients struggle with digital literacy, technical skills, and internet access [4,5]. Several patients showed interest in enrollment but faced challenges due to limited internet access and the capabilities of their cellular devices.

Additional challenges were associated with provider-level factors. Findings from our stakeholder engagement interviews and existing literature [16] indicate that time constraints for healthcare providers and staff limit their ability to integrate discussions about the portal into routine visits. Another challenge is staff turnover; it is inevitable in any workforce. Consequently, continuous training for both new and existing staff is essential to ensure consistent and accurate delivery of information related to patient portal enrollment and usage [12]. These findings align with the existing literature on digital health equity, emphasizing provider encouragement, patient education, and system-wide reforms as strategies for reducing disparities [20,21].

Another potential challenge to patient navigator-supported patient portal enrollment is sustainability and scalability. Initially backed by a grant from the National Institutes of Health (NIH), ongoing sustainability depends on investments from clinics and health systems. Some sites have incorporated digital health support roles into their staff, such as front-desk personnel helping with MyChart activation. Training existing medical assistants and front-desk staff to provide MyChart support may help maintain impact beyond grant-funded navigators’ roles. However, it is important to note that effective January 1, 2024, the Centers for Medicare & Medicaid Services (CMS) introduced new billing codes that allow Medicare to reimburse providers for patient navigation services. These codes cover various aspects of patient navigation, including assessing social determinants of health, providing caregiver training, and offering telehealth services. These funding and reimbursement initiatives aim to expand the role of patient navigators, particularly in underserved communities. By integrating navigators into healthcare teams, CMS seeks to improve patient engagement, reduce disparities in access to care, and enhance overall health outcomes.

Future research should investigate whether initial MyChart activation fosters long-term engagement. It is crucial to evaluate how frequently patients log in, whether they utilize features such as appointment scheduling and secure messaging, and whether portal usage enhances healthcare outcomes. For example, studies might assess whether heightened engagement improves medication adherence, fewer missed appointments, or more prompt preventive care. Understanding these trends will help determine whether patient navigation provides lasting benefits or requires additional support to maintain digital health participation.

In addition to monitoring engagement, qualitative research could offer valuable insights into patient and navigator experiences. Conducting interviews or focus groups with patients could explore their perceptions of the portal, the barriers they encounter post-enrollment, and which support mechanisms they found most beneficial. Navigators could also provide important perspectives on effective strategies, common challenges in assisting patients, and areas where further training or resources could enhance effectiveness. These insights would help refine patient navigation programs and inform best practices for boosting digital health literacy.

Comparative studies are also necessary to assess how navigator-assisted enrollment measures against other digital engagement strategies. Research should explore whether alternative methods, such as automated text or email reminders, provider encouragement during clinic visits, or financial incentives, are more effective or cost-efficient. Understanding how various strategies influence patient portal adoption and utilization will enable health systems to develop tailored, evidence-based interventions that effectively support diverse patient populations.

This study makes an important contribution to the literature on patient portal enrollment. However, its limitations should be acknowledged. The study was conducted in a Federally Qualified Health Center (FQHC) setting in a large urban setting with a predominantly African American and Latinx population. The findings may not be generalizable to other healthcare settings or populations. The study evaluated portal activation but not long-term usage (e.g., whether patients continued to log in, communicate with providers, or use online scheduling). Future research should track engagement for 6–12 months post-activation to assess longer-term engagement [22]. Without a robust control group, it is challenging to definitively attribute the observed increases in MyChart activation solely to the navigator program, as other health system-wide digital initiatives may have impacted MyChart activation trends. A randomized controlled trial (RCT) design could offer stronger causal evidence of navigator effectiveness.

## 3. Conclusion

This paper provides an overview of community engagement, pre-implementation planning, and the preliminary evaluation of a patient navigator-led patient portal delivery project. The evaluation results indicate that patient navigators are crucial in assisting patients in Federally Qualified Health Center (FQHC) settings in enrolling in MyChart. This approach could also benefit community health centers and hospitals that serve underserved and high-need populations. Health systems should consider incorporating navigation as a standard service and explore ways to sustain it. The Centers for Medicare & Medicaid Services (CMS) currently provides payment for patient navigators for key clinical activities. However, policymakers should continue to invest in programs that address digital health disparities in community care.

## Practice implications

Healthcare systems aiming to reduce digital health disparities in FQHCs can embed patient navigators into standard clinical processes. By offering real-time, culturally appropriate support, navigators assist patients in conquering technology barriers while enhancing trust in digital tools. In particular, the MyChart navigator model outlined here underscores how immediate, hands-on guidance can elevate enrollment rates, especially among underserved communities. Additionally, with new CMS billing codes covering navigation services, clinics have fresh opportunities to sustain and expand these interventions which may ultimately strengthen patient engagement and improve care coordination.

## References

[1] Garfan S, Alamoodi AH, Zaidan BB, Al-Zobbi M, Hamid RA, Alwan JK, Telehealth utilization during the Covid-19 pandemic: a systematic review. Comput Biol Med 2021;138:104878. 10.1016/j.compbiomed.2021.104878. 34592585 PMC8450049

[2] Predmore ZS, Roth E, Breslau J, Fischer SH, Uscher-Pines L. Assessment of patient preferences for telehealth in post-COVID-19 pandemic health care. J Am Med Assoc Netw Open 2021;4(12):e2136405. 10.1001/jamanetworkopen.2021.36405. PMC8637257 34851400

[3] Irizarry T, Shoemake J, Nilsen ML, Czaja S, Beach S, DeVito Dabbs A. Patient portals as a tool for health care engagement: a mixed-method study of older adults with varying levels of health literacy and prior patient portal use. J Med Internet Res 2017;19(3):e99. 10.2196/jmir.7099. 28360022 PMC5391436

[4] Ancker JS, Barrón Y, Rockoff ML, Hauser D, Pichardo M, Szerencsy A, Use of an electronic patient portal among disadvantaged populations. J Gen Intern Med 2011;26(10):1117–23. 10.1007/s11606-011-1749-y. 21647748 PMC3181304

[5] Dendere R, Slade C, Burton-Jones A, Sullivan C, Staib A, Janda M. Patient portals facilitating engagement with inpatient electronic medical records: a systematic review. J Med Internet Res 2019;21(4):e12779. 10.2196/12779. 30973347 PMC6482406

[6] Tieu L, Sarkar U, Schillinger D, Ralston JD, Ratanawongsa N, Pasick R, Barriers and facilitators to online portal use among patients and caregivers in a safety net healthcare system: a qualitative study. J Med Internet Res 2015;17(12): e275. 10.2196/jmir.4847. 26681155 PMC4704882

[7] Richwine C, Johnson C, Patel V. Disparities in patient portal access and the role of providers in encouraging access and use. J Am Med Inf Assoc 2023;30(2):308–17. 10.1093/jamia/ocad003. PMC9846679 36451262

[8] Matthews AK, Young Jung Min, Akufo J, Burke L, Dodd D, Donenberg G. Barriers to using a patient portal among low-income patient populations: a qualitative descriptive study. J Health Care Poor Under 2023;34(3):863–83. 10.1353/hpu.2023.a903052. 38015127

[9] Ali-Faisal P, Sobia F, Colella P, Tracey JF, Medina-Jaudes N, Benz Scott L. The effectiveness of patient navigation to improve healthcare utilization outcomes: a meta-analysis of randomized controlled trials. Patient Educ Couns 2017;100(3): 436–48. 10.1016/j.pec.2016.10.014. 27771161

[10] Nelson HD, Cantor A, Wagner J, Jungbauer R, Fu R, Kondo K, Effectiveness of patient navigation to increase cancer screening in populations adversely affected by health disparities: a meta-analysis. J Gen Intern Med 2020;35(10):3026–35. 10.1007/s11606-020-05927-2. 32700218 PMC7573022

[11] Freeman HP, Rodriguez RL. History and principles of patient navigation. Cancer 2011;117(S15):3537–40. 10.1002/cncr.26262. 21780088 PMC4557777

[12] Matthews AK, Steffen AD, Burke LA, Donenberg G, Duangchan C, Akufo J, The use of navigators to increase patient portal enrollment among patients in a federally qualified health care system (Special Issue) Ethn Dis 2023:117–25. 10.18865/ed.DECIPHeR.11. PMC11895548 38846728

[13] Kokorelias KM, Shiers-Hanley JE, Rios J, Knoepfli A, Hitzig SL. Factors influencing the implementation of patient navigation programs for adults with complex needs: a scoping review. 11786329211033267 Health Serv Insights 2021;14. 10.1177/11786329211033267. PMC8287353 34349519

[14] Graham ID, Logan J, Harrison MB, Straus SE, Tetroe J, Caswell W, Lost in knowledge translation: time for a map? J Contin Educ Health Prof 2006;26(1): 13–24. 10.1002/chp.47. 16557505

[15] Rabin BA, A citation analysis and scoping systematic review of the operationalization of the practical, robust implementation and sustainability model (PRISM). Implement Sci 2022;17(1):1–24. 10.1186/s13012-022-01234-3. 36153628 PMC9509575

[16] Ozkaynak M, Unertl K, Johnson S, Brixey J, Haque SN, Finnell JT, Clinical workflow analysis, process redesign, and quality improvement. In: Dixon BE, editor. Clinical Informatics Study Guide. Cham: Springer; 2022. p. 103–18. 10.1007/978-3-030-93765-2_8.

[17] Aghdam ZN, Rahmani AM, Hosseinzadeh M. The role of the Internet of things in healthcare: future trends and challenges. Comput Methods Prog Biomed 2021;199: 105903. 10.1016/j.cmpb.2020.105903. 33348073

[18] Alawiye TR. The impact of digital technology on healthcare delivery and patient outcomes. EHealth Telecom Syst Netw 2024;13(2):13–22. 10.4236/etsn.2024.132002.

[19] Sinha S, Garriga M, Naik N, Disparities in electronic health record patient portal enrollment among oncology patients. J Am Med Assoc Oncol 2021;7(6): 935–7. 10.1001/jamaoncol.2021.0540. PMC8033503 33830178

[20] Avdagovska M, Ballermann M, Olson K, Graham T, Menon D, Stafinski T. Patient portal implementation and uptake: qualitative comparative case study. J Med Internet Res 2020;22(7):e18973. 10.2196/18973. 32716308 PMC7427986

[21] Kruse CS, Bolton K, Freriks G. The effect of patient portals on quality outcomes and its implications to meaningful use: a systematic review. J Med Internet Res 2015;17 (2):e44. 10.2196/jmir.3171. 25669240 PMC4342639

